# Dietary diversity scores: an indicator of micronutrient inadequacy instead of obesity for Chinese children

**DOI:** 10.1186/s12889-017-4381-x

**Published:** 2017-05-12

**Authors:** Wenzhi Zhao, Kai Yu, Shengjie Tan, Yingdong Zheng, Ai Zhao, Peiyu Wang, Yumei Zhang

**Affiliations:** 1 Department of Nutrition and Food Hygiene, School of Public Health, Peking University Health Science Center, Xueyuan Road 38, Haidian District, Beijing, 100191 China; 2 Department of Epidemiology and Biostatistics, School of Public Health, Peking University Health Science Center, Xueyuan Road 38, Haidian District, Beijing, 100191 China; 3 Department of Social Medicine and Health Education, School of Public Health, Peking University Health Science Center, Xueyuan Road 38, Haidian District, Beijing, 100191 China; 4 Beijing Key Laboratory of Toxicological Research and Risk Assessment for Food Safety, Guangqu Road 37, Chaoyang District, Beijing, 100022 China

**Keywords:** Dietary diversity scores, Micronutrient, Nutrient adequacy, Obesity, Chinese children

## Abstract

**Background:**

Micronutrient malnutrition affects the well-being of both adults and children. Dietary diversity score (DDS) is a useful evaluation index with a relatively well-developed guideline by FAO. It’s meaningful to assess and predict inadequate micronutrient intakes using DDS in Chinese children, after ruling out the risk of obesity coming with more dietary diversity.

**Methods:**

Data for evaluation were extracted from the Nutrition Study of Preschool Children and School Children, which is a cross-sectional study covering 8 cities of China, including 1694 children in kindergartens and primary schools. This study applied DDS to Chinese children to test the validity for micronutrient inadequacy, and then explored the relationship between dietary diversity and obesity.

**Results:**

It reveals that dietary diversity varied with age and place of residence; the older ones and the ones living in rural areas tend to have poorer dietary diversity. Another discovery is that DDS is positively correlated with indicators of micronutrient adequacy, with a score of 6–8 indicating the lowest risk of micronutrient inadequacy in different groups of children. In our study population, dietary diversity is not related with obesity.

**Conclusions:**

Dietary diversity score is a valid indicator to evaluate micronutrient inadequacy in Chinese children, though there is still room for improvement of the method. Besides, the relationship between increase of dietary diversity and risk of obesity should be treated circumspectly.

## Background

Micronutrient malnutrition, which is also called hidden hunger, remains one of the most serious nutritional concerns in both children and adults, and in both developing and developed countries. While overweight and obesity have been a matter of public concern for decades in many countries, ranging from high-income countries to low- and middle-income countries [1, 2]. China is one of the world’s most economically dynamic nations with complex lifestyles, dietary patterns and nutrition transitions. Thus it has a double burden of both undernutrition and overnutrition, which is drawing more and more attention [3, 4]. It is worth noting that both micronutrient malnutrition and obesity have long-term influence on growth development and health of children.

In recent researches, there are several dietary diversity indicators, such as Dietary Diversity Scores (DDS) and Food Variety Score (FVS). DDS is based on food groups, which is more useful than indicators based on an individual food (e.g., FVS) in predicting nutrients adequacy, though the food groups in different researches vary a lot. Besides, attaching certain portion size requirements to DDS will improve the correlation between DDS and micronutrients intake, which has been proved by prior researchers [5, 6]. Since the simple DDS takes any amount of consumption as positive intake records for a certain category, it may overestimate the intakes of some groups. Research conducted in developing countries according to guidelines of FAO [6] or similar operation rules [5] all showed positive relationships between micronutrient adequacy and DDS, such as in non-breast-feeding Filipino children [7] or children in Mali who were under 60 month [8], and even in adults [9].

The inadequacy of iron, zinc, vitamin A and vitamin D is common among Chinese children according to related researches [10, 11]. In China, a 2002 National Survey reported that the prevalence of vitamin A inadequacy for 3–12 years old children was 9.3%, and that of Vitamin A marginal inadequacy was about 44.9% [10]. The prevalence of anemia for boys and girls were respectively 8.7 and 12.5% in urban areas, and 13.0 and 17.5% in rural areas, according to a national survey of 7–17 year olds [12]. Micronutrient malnutrition poses a serious threat to health. Every year Vitamin A inadequacy claims the lives of about 670,000 children under 5 and zinc inadequacy claims more than 450,000; and iron-inadequacy anemia results in 136,000 deaths of women and children [13, 14]. According to the estimation of WHO, Vitamin A supplement programs brought about a 70% reduction in childhood blindness; zinc programs reduced diarrhea incidence by 27%; folate programs prevented 50% neural tube birth defects [15].

Most of the micronutrients we need are from daily diet, and therefore it’s important to consume a balanced diet containing diverse food. Almost every country’s dietary guideline includes items about food diversity, given the fact that a variety of foods may ensure abundant amount of nutrients and other beneficial substances. More and more studies find positive association between dietary diversity and micronutrients adequacy, especially in children from developing countries [5–7, 15]. However, it’s possible that diverse diets provide adequate micronutrients with plenty of energy, or even more. Considering the increasing prevalence of obesity in children, circumspect decision should be made to improve their health. There are some researches focusing on relationship between dietary diversity and obesity, though the results vary a lot [16–18]. The latest Chinese Dietary Guidelines published in 2016 stress the importance of food dietary, and make a recommendation of consuming at least 12 food groups per day and 25 food groups per week. There are different ways of dividing food into different food groups, and 10 groups and 17 groups are defined in this paper according to Chinese Food Composition Tables and what children ate.

This study analyzed dietary diversity and micronutrient intakes of Chinese children of 3–12 years old, aiming to validate DDS as an indicator of micronutrient adequacy among children and to quantify an appropriate cut-off point as adequate micronutrient intake. Furthermore, the study analyzed the relationship between childhood obesity and dietary diversity to explore the double burden for Chinese children who were born and grew up in a transition period.

## Methods

### Subjects

Data were extracted from the Nutrition Study of Preschool Children and School Child, which was conducted from October 2011 to January 2012 in 8 cities of China. Details of the study were described elsewhere [19, 20]. The analysis included 1694 children in kindergartens and primary schools of 8 cities, aging between 3 and 12. Informed consents were obtained from their guardians prior to participation. The study was approved by the Ethical Committee of the Health Science Center at Peking University (NO.IRB00001052–11042).

### Dietary data

The food intake data were collected by four questionnaires, each containing a 24-h dietary record table. For every child in kindergartens, one questionnaire was completed by trained investigators to record what the children ate in kindergarten with the help of kindergarteners, and the other questionnaire was fulfilled by the same investigators to record what the children ate at home. For the children in primary school, another two questionnaires were used. All the personnels collecting the data were trained beforehand by professional researchers of the study uniformly.

We used a SAS program which was coded based on Chinese Food Composition Tables (CFCT) 2004 [21] and 2009 [22] (CFCT, National Institute of Nutrition and Food Safety, China CDC), and Standard Tables of Food Composition In Japan (2010) [23] and ingredient lists of common supplements to compute nutrients values, including energy, vitamin A, niacin, vitamin B6, riboflavin, vitamin B12, thiamine, vitamin C, folate, calcium, iron and zinc. Related publications and articles were referred to for information about phytate and oxalate in food. If the contents of phytate and oxalate of a certain type of food was missing, a similar food category was suggested.

The content of vitamin A in food was presented as retinol equivalent (RE) with a function of RE = vitamin A (μg) + β-carotene(μg)*1/6+ other pro-vitamin A carotenoids(μg)*1/12 [6]. For calcium, iron and zinc we computed absorbed amount by bioavailability adjustment, according to the absorption percentage of bioavailability. For calcium the absorbed percentages of different food types were determined by the contents of oxalate, 25% for roots, tubers, grains and legumes; 45% for fruits and vegetables; 5% for high oxalate food and 32% for the rest [6]. With reference to a list of high oxalate food provided by FAO, oxalate values were extracted from research articles to define the bioavailability percentage of fruits and vegetables with high oxalate content (high oxalate content meant >0.45 g oxalate per 100 g food). Absorbable iron was assumed to be 6% for plants and 11% for animal products. The absorbable zinc was calculated by the ratio of phytate to zinc for each food category (i.e., phytate: zinc molar ratio = ((mg phytate)⁄660)/(mg zinc⁄65.4)); if the ratio was over 18, the percentage of absorption was 30%, otherwise it was 22% [6].

### Dietary diversity scores

There were three indicators about dietary or food diversity calculated, namely two sets of dietary diversity scores based on 10 food groups (Dietary diversity scores, DDS) and 17 food groups (Seventeen-food-groups dietary diversity score, SDDS), and food variety score (FVS) counting all the food recorded in 24-h dietary records. According to the FAO protocol, DDS was calculated based on 10 food groups, as shown in Table 1 [6]. The child got one point if he or she consumed something at least once from a unique food group in 24-h dietary records, with a threshold of 10 g, which means one had to consume at least 10 g from a unique food group in the 24-h dietary records to get one point [6]. But for the group of oils and fats, the threshold was set at 2 g [6]. So the scoring range of DDS is from 1 to 9. To further explore the relationship between childhood obesity and dietary diversity or food variety, we also calculated segmented dietary diversity scores SDDS and FVS. SDDS ranged from 1 to 17, and its calculation was the same as DDS with a threshold of 10 g. As for FVS, we added up the number of different food items one consumed in 24-h dietary record. All the segmented food groups (showed in Table 1) and single food was defined according to China Food Composition.

**Table 1:** Food groups of 10-food-group dietary diversity scores (DDS) and 17-food-group dietary diversity scores (SDDS)

DDS		SDDS	
Food group	Note	Food group	Note
All starch staples	With a threshold of 10 g	Refined grains and cereals	With a threshold of 10 g
		Other cereals and grains	With a threshold of 10 g
		Starch tubers and roots	With a threshold of 10 g
Vitamin A-rich vegetables & fruit	With a threshold of 10 g	Vitamin A-rich dark-green leafy vegetables	With a threshold of 10 g
		Vitamin A-rich deep yellow/orange/red vegetables	With a threshold of 10 g
		Vitamin A-rich fruits	With a threshold of 10 g
All other fruits	With a threshold of 10 g	Vitamin C-rich vegetables	With a threshold of 10 g
All other vegetables	With a threshold of 10 g	Vitamin C-rich fruits	With a threshold of 10 g
		All other fruits & vegetables	With a threshold of 10 g
All legumes & nuts	With a threshold of 10 g	All legumes & nuts	With a threshold of 10 g
Oil and fat	With a threshold of 2 g	Oil and fat	With a threshold of 2 g
Meat, poultry, fish	With a threshold of 10 g	Meat, poultry, fish	With a threshold of 10 g
		Organ meat	With a threshold of 10 g
All dairy	With a threshold of 10 g	All dairy	With a threshold of 10 g
Eggs	With a threshold of 10 g	Eggs	With a threshold of 10 g
Other food	Exclude calculation	Desserts, candy, sugar-sweetened beverages and Puffing Food	With a threshold of 10 g
		Other food	With a threshold of 10 g

### Probabilities of adequacy

We referred to the FAO protocol to calculate probabilities of nutrients adequacy (PA) for a single day, based on distributions of each nutrient which were defined by the mean (i.e., estimated average requirements, EAR) and SD (standard deviation, i.e., the EAR multiplied by the distribution coefficient of variation, CV) [13]. We referred to Chinese Dietary Reference Intakes (DRIs) 2013 edition for EAR (i.e., estimated average requirements), and the CV was set to 10% of EAR for all nutrients except vitamin A (20%), niacin (15%) and zinc (25%) [7, 24]. The equation was PA = Probnorm [(estimated Child’s intake-EAR)/SD]; the Probnorm function calculated the probability of whether a child’s intake was above EAR. For iron, since its distribution wasn’t normal, we used the eq. PA = estimated Child’s intake/RNI (i.e, recommended nutrient intakes), with RNI extracted from Chinese DRIs. After we got PA of all the 11 micronutrients, we computed the mean probability of adequacy (MPA), applying equal weight to individual nutrients.

### Anthropometry data

Anthropometric information was included in an aforementioned survey. Children were weighed and measured without heavy clothing or shoes by trained investigators according to Chinese specification for children physical examination service, to the nearest 0.1 cm or 0.1 kg. For weight and height, calibrated scale (Suhong YES-S, RGZ-120; Jiangsu, China) was used. First, BMI was calculated for every child by dividing body weight (in kilograms) by height (in meters squared). LiHui’s study based on Chinese children was referred to to diagnose overweight and obesity by BMI [25]. Then related Z-scores were computed by WHO Anthro and WHO Anthroplus, including HAZ (Height for age Z-score), WAZ (Weight for age Z-score), WHZ (Weight for height Z-score) and BAZ (BMI for age Z-score).

### Statistical analysis

The analysis was conducted with SPSS version 15.0. *P*<0.05 was considered significant for all analysis. Descriptive analysis applied to continuous variables was showed as mean and SD, while categorical data were presented as frequency and percentage. Percentage of children who consumed different food groups according to DDS was sorted. Pearson’s correlation was tested by age group and residence between PA/MPA and DDS, with energy intake as a covariate. Sensitivity/ specificity analysis was performed to determine the DDS cut-off value for micronutrients inadequacy, using MPA as the gold standard. A certain MPA threshold was used to categorize children into low and high micronutrient intakes groups. Two MPA cut-off values of 0.50 and 0.75 were applied. Spearman’s correlation was tested between DDS, SDDS, FVS and BMI, Z-scores.

## Result

1694 children from kindergartens and primary schools were analyzed in this study, including 878 boys and 816 girls, and about 75% of them were from urban areas. The number of children who were ≤6 years was 983, and that of children who were >6 years was 711. The socio-demographic characteristics of investigated children were showed in Table 2.

**Table 2:** Demographic characteristics of investigated children

Characteristics	Note	Number, Percentage	
Nationality	Han nationality	1678, 96.9%	
	Minority nationality	54, 3.1%	
Per capita monthly income	≤1500 RMB	465, 27.7%	
	1501~3000 RMB	523, 31.1%	
	3001~6000 RMB	349, 20.8%	
	>6000 RMB	342, 20.4%	
		The mother of children	The Father of children
Educational level	Primary school or below	175, 10.1%	86, 5.0%
	Secondary education	1294, 74.8%	1289, 75.6%
	Bachelor degree or above	260, 15.0%	329, 19.3%
Occupation	Unemployed	608, 36.0%	129, 7.6%
	Office work	467, 27.6%	680, 40.2%
	Manual work	432, 25.6%	549, 32.5%
	Other work	183, 10.8%	332, 19.6%

The analysis of DDS, micronutrient intakes and PA/MPA of investigated children by age groups and residences was listed in Table 3. Age groups were determined by the fact of Chinese school age, and most of the investigated children between 6 to 7 years old were in kindergartens. The DDS distribution was significantly different between younger and older children, urban and rural children. Older children and children living in rural areas were more likely to get a lower score, and it’s significant that younger children (≤6 years) consumed more food groups than older children (>6 years), which meant that children in urban areas had better dietary diversity than those in rural areas. The micronutrients intakes and PA/MPA of investigated children shared the same trend, that was children of younger age group or living in rural areas were in better condition, for most micronutrients. It should be noticed that the intakes of minerals such as calcium, iron and zinc decreased by 70–90% after bioavailability adjusting, which had a big influence on the MPA. The MPA was 0.42 for children under 6, while 0.26 for children over 6. The significant difference was initially explained by the PAs of kinds of vitamin, while the PAs of minerals were both low in two groups.

**Table 3:** Distribution of DDS, nutrients intake and PA/MPA by age groups and residences

Items	Note	Total	Age group		Residence	
			≤6 years	>6 years	Urban	Rural
DDS	DDS	6.3 ± 1.5	6.8 ± 1.3***	5.6 ± 1.5***	6.4 ± 1.6***	5.8 ± 1.3***
Nutrients Intake	Energy(Kcal)	1432.7 ± 572.1	1448.7 ± 560.6	1410.6 ± 587.2	1438.4 ± 544.6	1415 ± 650.1
	Vitamin B1(mg)	0.7 ± 0.4	0.7 ± 0.4***	0.6 ± 0.3***	0.7 ± 0.4***	0.6 ± 0.3***
	Vitamin B2(mg)	0.8 ± 0.4	0.8 ± 0.4**	0.7 ± 0.4**	0.7 ± 0.4**	0.8 ± 0.5**
	Niacin(mg)	10.2 ± 5.7	10.2 ± 5.5	10.1 ± 6.1	10.9 ± 5.8***	7.9 ± 4.9***
	Vitamin B6(mg)	0.6 ± 0.3	0.6 ± 0.3***	0.5 ± 0.3***	0.6 ± 0.3***	0.4 ± 0.3***
	Folate(μg)	149.0 ± 98.5	152.9 ± 86.6	143.7 ± 112.8	142.7 ± 89.3***	168.5 ± 120.7***
	Vitamin B12(μg)	2.4 ± 3.8	2.4 ± 3.4	2.3 ± 4.2	2.7 ± 4.2***	1.4 ± 1.7***
	Vitamin C(mg)	58.1 ± 46.2	59.3 ± 41.7	56.5 ± 51.8	58.5 ± 45.7	56.9 ± 47.7
	Vitamin A(μg RE)	259.8 ± 221.9	285.0 ± 211.9***	224.9 ± 230.6***	280.3 ± 236.5***	196.3 ± 152.3***
	Absorbed Calcium(mg)	109.3 ± 74.8	112.7 ± 80*	104.4 ± 66.6*	115.4 ± 75.4***	90.2 ± 69.6***
	Absorbed Iron(mg)	1.0 ± 0.6	1.0 ± 0.5	1.0 ± 0.7	1.0 ± 0.6	1.0 ± 0.5
	Absorbed Zinc(mg)	2.1 ± 1.0	2.1 ± 0.9	2.1 ± 1.1	2.2 ± 1.0***	1.9 ± 0.9***
	Calcium(mg)	341.5 ± 228.5	353.1 ± 245.9*	325.4 ± 201.1*	356.5 ± 228.7***	294.9 ± 221.6***
	Iron(mg)	14.4 ± 8.1	14.4 ± 6.9	14.4 ± 9.6	14.2 ± 7.9	15.0 ± 8.9
	Zinc(mg)	7.4 ± 3.5	7.5 ± 3.3	7.3 ± 3.6	7.7 ± 3.5***	6.7 ± 3.3***
PA/MPA	PA(Vitamin B1)	0.39 ± 0.45	0.55 ± 0.46***	0.17 ± 0.33***	0.42 ± 0.46***	0.28 ± 0.39***
	PA(Vitamin B2)	0.50 ± 0.46	0.64 ± 0.43***	0.31 ± 0.42***	0.52 ± 0.45	0.45 ± 0.46
	PA(Niacin)	0.66 ± 0.42	0.76 ± 0.37***	0.51 ± 0.45***	0.73 ± 0.40***	0.44 ± 0.42***
	PA(Vitamin B6)	0.31 ± 0.42	0.43 ± 0.45***	0.14 ± 0.31***	0.35 ± 0.44***	0.17 ± 0.33***
	PA(Folic Acid)	0.32 ± 0.42	0.44 ± 0.44***	0.15 ± 0.33***	0.31 ± 0.42	0.34 ± 0.44
	PA(Vitamin B12)	0.65 ± 0.46	0.76 ± 0.41***	0.50 ± 0.47***	0.72 ± 0.43***	0.44 ± 0.48***
	PA(Vitamin C)	0.53 ± 0.47	0.64 ± 0.46***	0.36 ± 0.45***	0.53 ± 0.48	0.50 ± 0.47
	PA(Vitamin A)	0.33 ± 0.41	0.47 ± 0.42***	0.15 ± 0.30***	0.38 ± 0.42***	0.19 ± 0.33***
	PA(Calcium)	0 ± 0.03	0 ± 0.04*	0 ± 0*	0 ± 0.02	0 ± 0.05
	PA(Iron)	0.64 ± 0.26	0.66 ± 0.25**	0.61 ± 0.27**	0.65 ± 0.26**	0.60 ± 0.28**
	PA(Zinc)	0.05 ± 0.14	0.08 ± 0.17***	0.02 ± 0.07***	0.06 ± 0.15***	0.02 ± 0.06***
	MPA	0.39 ± 0.26	0.49 ± 0.26***	0.26 ± 0.19***	0.42 ± 0.27***	0.31 ± 0.22***

*PA* probabilities of nutrients adequacy, *MPA* mean probability of adequacy; *, *P*<0.05; **, *P*<0.01; ***, *P*<0.001

**Fig. 1:**
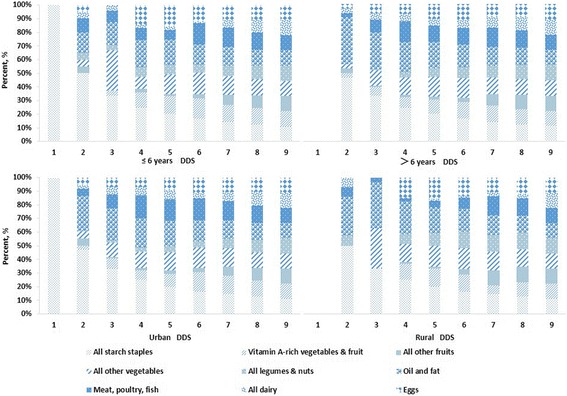
Probability of investigated children consuming food groups by DDS, divided by age groups and residences

As shown in Fig. 1, the probability of every food group being consumed was different and changed with the increase of DDS, and for children who got a DDS of 1, they just ate all starch staples by 100 % that day. The difference between age groups and places of residence was significant. Groups such as all starch staples, oils and fats, other vegetables, meat, poultry, fish were the most frequently consumed food.

**Fig. 2:**
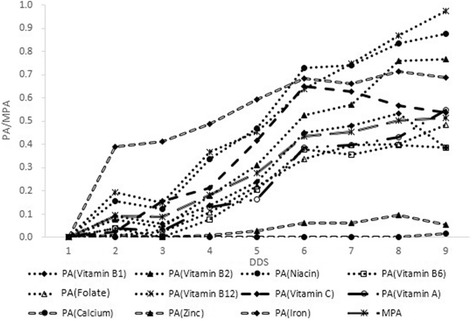
PA of micronutrients and MPA at different levels of DDS from investigated children

The change of PA/MPA by DDS was shown in Fig. 2. For all vitamins, the PA tended to increase with higher score in DDS, and PA of vitamin B12 and C were almost linearly associated with DDS. For minerals, however, the trends were different, and the lines showing PA of minerals changing with DDS were flat and gentle. We further analyzed the correlations between PA/MPA and DDS, and found that only the correlation between PA of absorbed zinc and DDS was non-significant. The correlation coefficient between MPA and DDS was 0.372. When stratifying investigated children by age (≤6 years and>6 years), the correlations coefficient between MPA and DDS were 0.148 and 0.338, respectively. While when the investigated children were stratified by place of residence (Urban and Rural area), the correlation coefficient between MPA and DDS were 0.349 and 0.296. Though the FAO protocol was designed to assess children under 6, there was no advantage in our “matched” population.

**Fig. 3:**
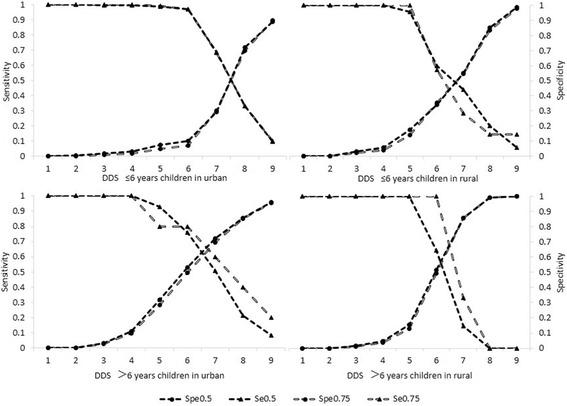
Sensitivity and specificity of DDS for 2 different cut-off points of MPA for investigated children by age and residence

Figure 3 showed sensitivity and specificity of DDS as an indicator to predict or evaluate micronutrient intake adequacy of investigated children, with MPA as the golden standard. Sensitivity indicated low DDS correctly reflected the percentage of investigated children truly at risk of micronutrients inadequacy (low MPA). Specificity showed the percentage of investigated children with no risk of micronutrient inadequacy (high MPA) who were defined with higher DDS properly. We defined the intersection of sensitivity and specificity lines as the DDS cut-off value to classify the two groups of micronutrients inadequacy and adequacy. It’s reasonable that intersection of larger MPA corresponded to higher DDS. Though the two groups of lines nearly overlapped in urban children ≤6 years, and the intersection of larger MPA corresponded to lower DDS in rural children ≤6 years. Comparing the DDS cut-offs in different age-residence groups, almost all the intersections of MPA = 0.75 were around 7 or 8, except for that of rural. So if one investigated child in urban areas got 7or 8 in DDS system, we may worry less about him for risk of micronutrient inadequacy. But for children in rural areas, we should lower the DDS to 6 to determine they were in danger of micronutrient inadequacy or not.

Then we conducted correlation analysis of food diversity and anthropometric data, especially on indicators about overweight and obesity. The descriptive analysis was shown in Table 4. Just as DDS, there was significant difference between 2 age groups and places of residence in SDDS and FVS, and the trend was that children of younger age group and urban areas consumed more food items or groups. For growth indicators and obesity rate, there was only significant difference between two age groups in BMI, but when comparing children from urban areas and rural areas, their means of BMI, WHZ, WAZ, BAZ and obesity rate were all significantly different. The correlation analysis of dietary diversity indicators and growth indicators were shown in Table 5. It’s interesting that in children >6 years old, dietary diversity indicators were negatively correlated with BMI and BAZ, which was just opposite to those of children ≤6 years old.

**Table 4:** Distribution of SDDS, FVS, Z-scores and rate of overweight/obesity by age groups and residences

Item	Note	Total	Age group		Residence	
			≤6 years	>6 years	Urban	Rural
Food diversity	SDDS	7.5 ± 2.1	8.4 ± 1.7***	6.2 ± 1.8***	7.7 ± 2.2***	6.9 ± 1.6***
	FVS	13.2 ± 5.3	15.7 ± 4.7***	9.6 ± 3.7***	14.1 ± 5.5***	10.3 ± 2.8***
Growth indicator	BMI	16.6 ± 8.1	15.8 ± 3.0***	17.3 ± 10.9***	17.0 ± 9.2***	15.2 ± 2.5***
	WHZ	0.2 ± 1.5	0.2 ± 1.5		0.3 ± 1.5**	−0.2 ± 1.1**
	WAZ	0.3 ± 1.3	0.3 ± 1.2	0.3 ± 1.3	0.4 ± 1.3***	−0.1 ± 1.1***
	HAZ	0.3 ± 1.2	0.3 ± 1.2	0.2 ± 1.2	0.3 ± 1.2	0.3 ± 1.2
	BAZ	0.1 ± 1.6	0.2 ± 1.6*	0.1 ± 1.4*	0.4 ± 1.5***	−0.6 ± 1.4***
Obesity rate	Overweight and obesity	375, 22.1%	197,2 0.0%	178, 25.0%	344, 26.9%***	31, 7.5%***
	Obesity	154, 9.1%	86, 8.7%	68, 9.6%	142, 11.1%***	12, 2.9%***

*SDDS* Seventeen-food-groups dietary diversity score, *FVS* food variety score, *HAZ* Height for age Z-score, *WAZ* Weight for age Z-score, *WHZ* Weight for height Z-score, *BAZ* BMI for age Z-score; −-, not applicable; *, *P* ˂0.05; **, *P* ˂0.01; ***, *P* ˂0.001

**Table 5:** Correlation analysis of dietary diversity indicators and growth indicators

Variable	DDS			SDDS			FVS		
	Total	≤6 years	>6 years	Total	≤6 years	>6 years	Total	≤6 years	>6 years
BMI	−0.109**	0.048	−0.085*	−0.113**	0.158**	−0.124	−0.111**	0.186**	-0.135**
WHZ	-0.037	−0.037	--	0.118**	0.118**	--	0.164**	0.164**	--
WAZ	0.042	0.037	0.030	0.084**	0.151**	−0.042	0.082**	0.137**	-0.037
HAZ	0.067**	0.012	0.078*	0.060*	0.066*	0.002	0.048*	0.015	0.017
BAZ	0	0.051	−0.076*	0.036	0.166**	−0.120*	0.048*	0.193**	-0.130**

*DDS* dietary diversity score of 9 food groups, *SDDS* Seventeen-food-groups dietary diversity score, *FVS* food variety score, *HAZ* Height for age Z-score, *WAZ* Weight for age Z-score, *WHZ* Weight for height Z-score, *BAZ* BMI for age Z-score; −-, not applicable; *, *P* ˂0.05; **, *P* ˂0.01

**Fig. 4:**
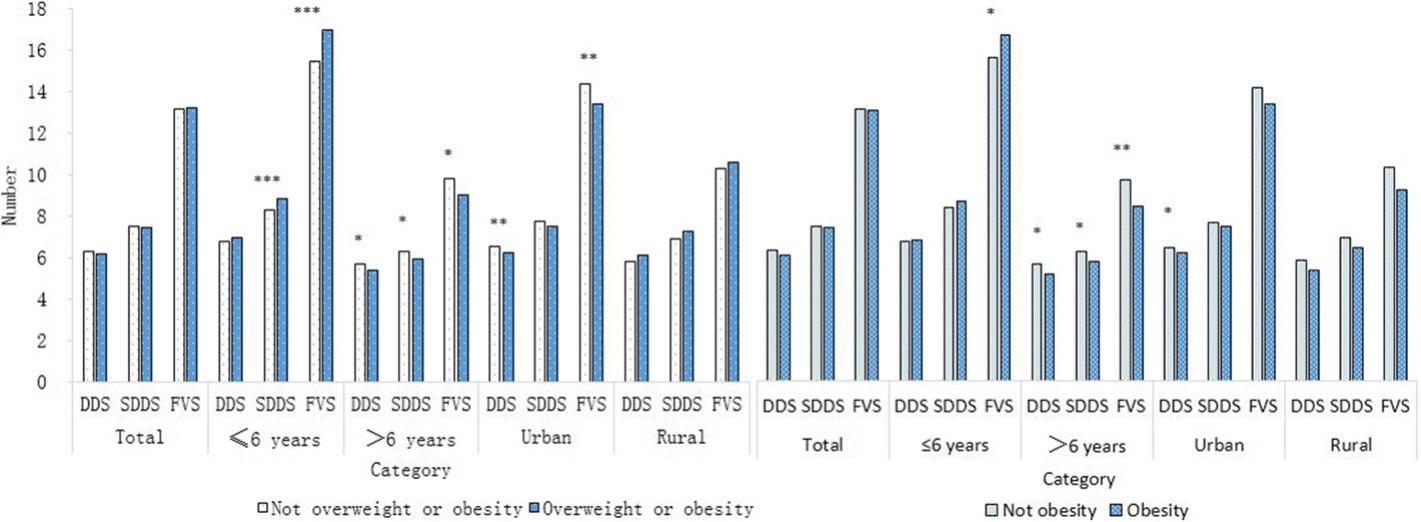
Comparison of food diversity indicators between children with obesity problem and without, Definition: DDS, dietary diversity score of 9 food groups; SDDS, Seventeen-food-groups dietary diversity score; FVS, food variety score; *, *P*<0.05; **, *P*<0.01; ***, *P*<0.001

Furthermore, we compared 3 food diversity indicators between children who had obesity problem (overweight or obesity) and that who did not. The results were shown in Fig. 4. In total, we failed to find significant relationship between food diversity indicators and obesity status, too. But in different age groups and residence groups, there were significant differences. When children who were overweight or obesity were defined as obese children, obesity children in the younger children group got higher SDDS and FVS, while obesity children in the older children group got lower DDS, SDDS and FVS. When only children who were obesity were defined as obesity children, the results were similar: obesity children in urban areas got lower DDS than non-obesity children.

## Discussion

Lack of dietary diversity is common among underserved population from developing countries, for the reason that their diets are largely based on starchy staples, with little or no animal products and few fresh fruits and vegetables. The plant-based diets will result in inadequate mineral micronutrients intake or poor absorption. In our study, the investigated children consume 6 food groups on average, and the older children consume fewer groups than the younger ones. The most frequently consumed food groups are starch staples, oils and fats, other vegetables, meat, poultry, fish, and dairy, while the groups of legumes, pulses and nuts, dairy and other fruits are less likely to be consumed. Compared with studies conducted in other developing countries such as Kenya, the Philippines, Mali and South Africa [7–9, 26], the studied sample consumed more food groups.

In our study the older children tend to get a lower DDS score, which is contrary to common sense that older children should have more chance and better appetite to eat different kinds of food. In China, people pay more attention on children, normally it has been suggested that there should be one dietician at least in the kindergarten who is responsible for the children’s diets. There are several kinds of weekly dietary plan made according to Chinese dietary guidelines for different age of children in kindergartens which are administrated by Health Bureau. And the diets for kindergarten children are thought highly of and pleased by children and their parents. However, in primary school, things are more different, and there are only a few of schools making their dietary plan for students. The primary school children also eat lunch at school, while their dietary habits differ a lot, and related studies show that a lot of students do not like lunches supplied in school and food waste is common in students [27, 28]. The shift of the guardians’ focus from children’s growth to their study, and the formation of independent selectivity may be responsible for the trend. Children in rural areas have worse dietary diversity than those in urban areas, which may be explained by household socioeconomic factors [5, 18].

For children, micronutrients do play an important role in their growth and development. In DDS system, PA acts as an indicator of intake adequacy, while the limit is being defined subjectively according to researchers [5]. There are positive correlations between PA of most micronutrients and DDS, meaning the more food groups one consumed, the lower risk of him being exposed to micronutrient inadequacy. The Pearson’s correlation between DDS and MPA is statistically significant, as has been backed by similar researches, such as those in Philippines (0.44), Mali (0.39) and Kenya (0.32) [7–9]. For vitamins, especially vitamin B12 and vitamin C, consuming more food groups will ensure adequate intake. However, for zinc and calcium, consuming more food groups seldom affects the absorbed dose, suggesting that we should pay attention to lower bioavailability of minerals. The fact that after bioavailability adjustment, the content of mineral micronutrients decreases a lot, suggests that diet mostly based on plants is barely enough to provide balanced nutrients [29, 30]. There are few studies or official documents focusing on content of “disturbed substances” in food materials and their influence on nutriment intakes in China and the disturbed substances involve all the chemical substances that will obstruct the absorption of minerals in alimentary canal, such as phytate, oxalate and polyphenols in plants. It is time we take the natural disturbance seriously and draw on related studies in making our guidelines about dietary or nutrition.

There are two most important steps we did to calculate PA of minerals, supplementing the content of “disturbed substances” in plant food for bioavailability adjustment and referring to the Chinese DRIs. As mentioned earlier, we refer to a lot of studies to get the content of phytate and oxalate in different food. However, the content of phytate and oxalate is greatly affected by planting environment and processing method in plant [31, 32]. When calculated with Chinese DRIs, the PA is inevitable to deviate from the reality of Chinese children. Though the trend of PA is similar to that of DDS, that is PA and MPA of younger children and children in urban areas are higher. So the combination of DDS and PA/MPA shows that in our study population children in kindergarten and children in urban areas are in better micronutrient condition.

In validation tests of DDS and adequate micronutrient intake, the point of intersection of sensitivity and specificity lines indicates the distinguishable score, separating children with high risk of micronutrient inadequacy from the ones with low risk. As a next step, we could just use DDS cut-off to screen children at high risk. Our research provides different cut-offs according to children’s age and place of residence. For younger children in urban areas, one has to consume nearly 8 food groups to avoid risk of micronutrient inadequacy; while younger children in rural areas and all the older children should consume 6 or 7 food groups to be free from the risk. It’s reasonable that in the population with less dietary diversity, we should lower the threshold of DDS distinguishing higher risk of micronutrient inadequacy.

In our study population, we failed to find the relationship between DDS and overweight or obesity, nor that between SDDS and overweight or obesity, nor that between FVS and overweight or obesity, similar to previous studies [16, 17]. Though in other researches, they do find relationship between dietary diversity and obesity [33, 34]. The positive results in subgroup analysis are in accordance with the two opinions in part. In children of higher socio-economic status or living in urban areas, obesity children tend to have low dietary diversity [16]. However, the opposite trend of DDS, SDDS and FVS changing with obesity status in the two age groups called for more in-depth researches. The dilemma is that, in younger children the increase of dietary diversity improves micronutrient status without bringing in risk of obesity, and the older children face a more complex situation-higher dietary diversity coming with better micronutrient status but higher risk of obesity, according to the preliminary results. There is a lot of work to do to explain and solve the problem.

One of the limitations of our study is that we collected dietary information just for 24 h, and it is just representative for daily life in kindergartens or schools, which will affect the validity of PA/MPA and DDS. We should prolong the data collection period in future research, such as a 3-day dietary recall including workdays and off days. Furthermore, we need to update our food composition table based on Chinese food, adding the contents of some important nutrients and substances disturbing absorption. Guidelines or standards from other countries cannot meet all the needs because the dietary is so different. Another shortage is that the guidelines of FAO are applicable for non-breastfeeding children aged between 2 and 6 years old, but we expand the recommended population, which may affect the validity of the sensitivity and specificity estimate for older children.

Our study is the first one to evaluate Chinese children’s dietary diversity with the FAO guidelines. We found positive correlation between DDS and MPA/PA, but failed to find that of DDS/SDDS/FVS and obesity status. The results of different subgroups varied greatly, and we should evaluate the nutrition status of different populations separately. The thresholds of DDS provided reference for future screening projects [11]. Currently, there is no quick and easy way to determine the micronutrient adequacy of the diet of children in China, and detection of children’s blood trace elements is an expensive but not necessary test for some nutrients such as calcium, although it is formal in some hospitals. The health status of children under school age are managed by community health service centers and each child has several times of health examination according to principles of the Health Bureau. So the development and promotion of DDS as a screening tool in hospitals and health service centers is helpful for doctors to assess nutritional status of children.

## Conclusions

Dietary diversity score is a valid indicator to evaluate micronutrient inadequacy in Chinese children, though there is still room for improvement of the method. Besides, the relationship between increase of dietary diversity and risk of obesity should be treated circumspectly in Chinese children.

## Abbreviations

BAZ: BMI for age Z-score
BMI: Body mass index
CFCT: Chinese food composition Tables
CV: Coefficient of variation
DDS: Dietary diversity score
DRIs: Dietary reference intake
EAR: Estimated average requirements
FVS: Food variety score
HAZ: Height for age Z-score
MPA: Mean probability of adequacy
PA: Probabilities of nutrients adequacy
RE: Retinol equivalent
RNI: Recommended nutrient intakes
SD: Standard deviation
SDDS: Seventeen-food-groups dietary diversity score
WAZ: Weight for age Z-score
WHZ: Weight for height Z-score
